# Analysis of the unique structural and physicochemical properties of the DraD/AfaD invasin in the context of its belonging to the family of chaperone/usher type fimbrial subunits

**DOI:** 10.1186/1472-6807-11-25

**Published:** 2011-05-16

**Authors:** Rafał J Piątek, Piotr Bruździak, Beata M Zalewska-Piątek, Marek A Wojciechowski, Justyna M Namieśnik, Józef W Kur

**Affiliations:** 1 Department of Microbiology, Gdańsk University of Technology, ul. Narutowicza 11/12, 80-233 Gdańsk, Poland; 2 Department of Physical Chemistry, Gdańsk University of Technology, ul. Narutowicza 11/12, 80-233 Gdańsk, Poland; 3 Department of Pharmaceutical Technology and Biochemistry, Gdańsk University of Technology, ul. Narutowicza 11/12, 80-233 Gdańsk, Poland

## Abstract

**Background:**

DraD invasin encoded by the dra operon possesses a classical structure characteristic to fimbrial subunits of the chaperone/usher type. The Ig-fold of the DraD possesses two major characteristics distinguishing it from the family of fimbrial subunits: 1) a distortion of the β-barrel structure in the region of the acceptor cleft, demonstrated by a disturbance of the main-chain hydrogen bonds network, and 2) an unusually located disulfide bond connecting B and F strands - the localization exclusively observed in the subfamily of DraD/AfaD-type subunits.

**Results:**

To evaluate the influence of the DraD-sc specific structural features on its stability and mechanism of thermal denaturation, a series of DSC and FT-IR denaturation experiments were performed giving following conclusions. 1) The DraD-sc is characterized by a low stability (standard Gibbs free energy and enthalpy of unfolding of 18.4 ±1.4 kJ mol^-1 ^and 131 ±25 kJ mol^-1^, respectively) that contrasts strongly with almost infinite stability of the described previously DraE-sc fimbrial protein. 2) The DraD-sc unfolds thermally according to the two state equilibrium model, in contrast to the irreversible kinetically controlled transition of the DraE-sc. 3) The DraD specific disulfide bond is crucial at the folding stage and has little stability effect in the mature protein.

**Conclusions:**

Data published so far emphasize unique biological properties of the DraD invasin as fimbrial subunit: a chaperone independent folding, an usher independent surface localization and the possibility to exist in two forms: as unbound subunits and as loosely bound at fimbrial tip.

Presented calorimetric and FT-IR stability data combined with structural correlations has underlined that the DraD invasin is also characterized by unique physicochemical and structural attributes in the context of its belonging to the family of fimbrial subunits.

## Background

Adhesive structures of the chaperone\usher type encoded by Gram-negative bacteria are conserved at the level of an operon organization, a mechanism of biogenesis, and a molecular structure. The key feature of these organelles are their building blocs - protein subunits characterized by the defective immunoglobulin-like structure. In contrast to the classical Ig-fold, a closed β-barrel composed of 7 β-strands, these subunits are missing the seventh strand and expose the arising hydrophobic cleft to a solvent. In result, these fimbrial subunits need to interact with a specific chaperone to fold properly. According to the donor strand complementation reaction (DSC), a subunit forms with a chaperone the binary complex in which the groove is completed by the G1 donor strand of the chaperone [1-3]. In adhesive organelles the acceptor cleft of a 'n' subunit is supplemented by the N-terminal donor strand G_d _of a consecutive 'n+1' subunit [4,5]. Formation of a functional linear adhesive structure occurs through the outer membrane usher protein according to the donor strand exchange reaction (DSE) [6,7]. This mechanism of biogenesis is very well investigated at the structural and energetic level especially for type 1, P pili of *E. coli *and the capsular F1 antigen of *Yersinia pestis *(for review see: [8,9]).

Adhesive organelles of the chaperone/usher system belong to the most stable protein structures ever described [10,11]. The observed resistance to unfolding is an outcome of the structural stability of protein subunit modules. The denaturation of a subunit results in a destruction of the adhesive structure. Hence, the self-complemented subunits that possess a C-terminally fused specific G_d _donor strand (denoted by the -sc suffix) are minimal approximations of these adhesive organelles. The data published so far permit to distinguish two general mechanisms of these subunits stabilization that may cooperate in the proteins. The first is based on a thermodynamic stability and is denoted by a high Gibbs free energy of unfolding and an enthalpy of unfolding. Most proteins possess the standard unfolding free energy in the range of 20 - 60 kJ mol^-1^. This 'energetic window' is a natural consequence of the protein metabolism in the living organism, and the occurrence of proteins with higher values of ΔG is rare and unfavorable from physiological point. The self-complemented Caf1-sc subunit of the capsular F1 adhesin is stabilized by the unfolding free energy ΔG_37°C _of 70-80 kJ mol^-1^. This corresponds to the equilibrium constant K_unfold _of magnitude 10^-12 ^- 10^-14 ^[5]. This value implies an almost infinite stability to the F1 capsular antigen at the physiological temperature, even in spite of the irreversibility of adhesive organelles unfolding. The Caf1 protein does not possess any special structural motif responsible for its high thermodynamic stability. It is rather an effect of a global optimization of the interactions that stabilize the Ig-barrel structure [4,5]. The second mechanism is based on a subunit kinetic stability denoted by a high energy of activation of the unfolding stage [12,13]. The self-complemented AfaE-sc subunit of Afa-III adhesin (98% identity to DraE subunit of Dr fimbriae) is characterized by the moderate unfolding free energy ΔG_25°C _of 45.1 ± 1.5 kJ mol^-1 ^[14]. Although the DraE-sc is effectively protected from the unfolding by the activation barrier E_a _of 463.5 ± 20.8 kJ mol^-1 ^and corresponding rate constant 10^-17^s^-1^, which results in its unfolding half-life time of 10^8 ^years at 25°C [15]. DraE and many other fimbrial subunits are characterized by a disulfide bond, which is uniquely localized compared to other members of the Ig-type protein superfamily, as it connects the A and B strands. Our previous work showed that the DraE-sc protein with cysteine residues changed to alanine lost its kinetic stability [13]. This suggests that such a localization of the disulfide bond is important for generation of the kinetic stability of the fimbrial proteins.

**Figure 1:**
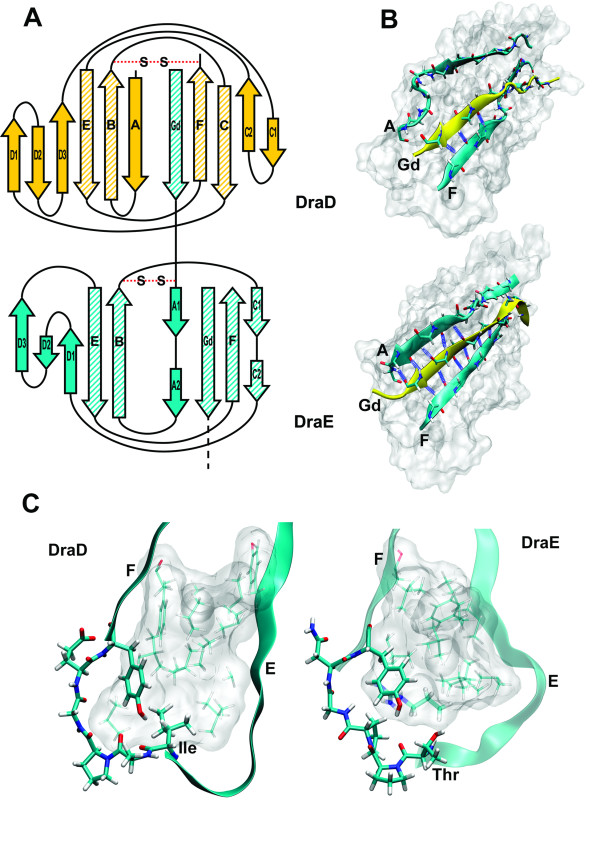
**Structural differences between DraD/AfaD and DraE/AfaE fimbrial subunits**. **(A) **Topology diagram of DraD-DraE tip complex. The core forming strands are striped, the S-S denotes disulfide bond. **(B) **Comparison of DraD-sc and DraE-sc acceptor cleft area. In both proteins A and F strands forming the groove as well as the donor G_d _strand are shown as ribbons. Backbone hydrogen bonds participating in the β-barrel formation are drawn as blue springs. **(C) **Tyrosine corner joining E and F strands. Residues of the canonical tyrosine corner motif are drawn as thick sticks while side chains of residues comprising the hydrophobic core of the protein as thinner sticks. Arg16 moiety completely buried in the core of DraE protein is drawn with thicker line. The figures were generated with VMD program (Theoretical and Computational Biophysics Group, Beckman Institute for Advanced Science and Technology, University of Illinois at Urbana-Champaign) [42].

The *dra *and the *afa-III *operons encode FGL-type polyadhesins: the Dr fimbriae composed of DraE proteins and the Afa-III afimbrial polymers composed of AfaE-III subunits (98% identity to the DraE), respectively [16]. These adhesin proteins are responsible for bacterial attachment to the host urinary epithelia via interaction with: the Dr^a ^blood-group antigen presented on the CD55/decay-accelerating factor (DAF) [17,18], the carcinoembryonic antigen (CEA)-related cellular adhesion molecules [19] and the 7S domain of the basement membrane protein type IV collagen [20,21]. The *dra *and *afa-III *gene clusters encode also second minor fimbrial subunits: the DraD and AfaD proteins (100% identity), respectively. These proteins are denoted as putative invasins particles crucial in the promotion of effective bacterial internalization [16,22]. Although this function and definition of specific receptor for DraD/AfaD are under debate [23,24]. In the context of the presented general conservation of chaperone\usher type fimbrial subunits properties of the family of the DraD/AfaD-like invasin subunits are very interesting [16]. The crystallographic and NMR structures of the DraD/AfaD invasin show that they possess the classic Ig-like fold with a characteristic hydrophobic groove as a consequence of the missing seventh G strand [14,25]. A specific feature of these invasins is the lack of N-terminal donor strand G_d _crucial for the donor strand exchange reaction (DSE) (Figure 1A). In consequence, the DraD/AfaD subunit may only exist at the tip of the fimbrial structure, although there are no direct evidences of interactions between the DraD invasin and the DraE adhesin. The structural comparison between DraD-C-His [25], AfaD-sc [14], AfaDE-sc [14] and AfaE-sc [26] proteins reveals the unusual properties of the invasin acceptor cleft. In the DraD-C-His structure of a homodimer the acceptor pocket of one subunit is complemented by a nonspecific C-terminal fusion peptide of the second subunit. The peptide does not penetrate into the hydrophobic groove and forms hydrogen bonds only with the F strand and does not with the A strand. Hence, the cleft of the DraD-C-His is opened to the solvent and the β-barrel is 'unzipped' [25]. In the AfaD-sc and AfaDE-sc proteins the acceptor cleft of the invasin is filled in by a specific G_d _donor strand of the AfaE subunit introduced as a C-terminal fusion peptide, or as an extension of AfaE-sc fused to the AfaD by the DNKQ linker, respectively. Surprisingly, in these structures the acceptor cleft of the invasin is opened to the solvent as in the DraD-C-His case (Figure 1B) [14]. On the contrary, in the AfaE-sc adhesin structure the G_d _donor strand precisely interacts with the acceptor pocket participating in a formation of the protein hydrophobic core (Figure 1B). This phenomena may explain the instability of the DraD-DraE protein complex and may result in a dissociation of the DraD from the fimbrial tip [22,27].

Another structural element distinguishing DraD invasin from the other fimbrial subunits is localization of disulfide bond. In contrast to the canonical Ig-fold, position of the disulfide bridge in many fimbrial subunits is unusual since it joins two adjacent strands of the same sheet of the β-sandwich, namely the beginning of A strand with the end of B strand [13]. Beside the atypical position in the sequence also the spatial location of the bridge is unusual since it is placed at the top of the β-barrel unlike in the canonical Ig-structures, where the disulfide bond joins the strands in the middle thus positioning itself in the hydrophobic core center of the protein. Figure 1A represents as an example the localization of a disulfide bond in the DraE fimbrial subunit. In the DraD protein the disulfide bond links two strands belonging to the opposite sheets, namely the B and F strands and hence resembles the canonical Ig-fold location (Figure 1A). Despite the different connectivity compared to DraE (and other subunits with disulfide bond), the location of the disulfide near the top of the β-barrel in the three-dimensional structure of DraD resembles the localization in DraE. It does so by linking the end of B strand with the end of F strand while the usual canonical Ig-fold location of the disulfide link is B3 F3. Interestingly, in the family of DraD/AfaD-like invasins the potential disulfide bonds homologous to that identified in the structure of DraD/AfaD occurs in proteins encoded only by the *afa *and *afa-*like operons. The putative invasins SafD and SefD from *Salmonella *do not possess two cysteines and thus do not have a disulfide bond.

The Cota et al. [14] using equilibrium denaturation experiments denoted low stability of the AfaD-sc protein and determined its unfolding free energy of 17.9 ± 6.8 kJ mol^-1^. In this paper we evaluated, by means of the DSC calorimetry and FT-IR spectroscopy, the influence of the unusual structural properties of the Ig-like fold of DraD/AfaD invasin on its stability and the mechanism of thermal unfolding. Two major structural elements specific to the DraD in the context of its belonging to the superfamily of fimbrial subunits are carefully correlated with experimental data. 1) The DraD protein possesses the disulfide bond connecting the B and F strands that is unique to the family of fimbrial subunits. This renders that the DraD invasin may be used in some extent as an additional natural verification of previously presented thesis that the disulfide bond connecting A and B strands is responsible for the observed high kinetic stability of the DraE-sc [13]. 2) The network of the inter-strands main-chain hydrogen bonds stabilizing the β-barrel structure of the DraD is defective in the region of acceptor cleft. This contrasts with the general view of precise docking of G_d _donor strands in fimbrial subunits observed so far. In our experiments we used a recombinant DraD-sc protein with the histidine tag at the N-terminus and self-complemented at the C-terminus by attaching donor strand sequence from DraE protein. This construct of DraD protein is a minimal model of fimbrial tip complex DraD-DraE. This protein is identically constructed as the DraE-sc protein described previously [15] and analogically to the other fimbrial self-complemented subunits. This strategy permitted us to perform a straight comparison between parameters characterizing the Ig-like structure of DraD-sc with that of the other fimbrial subunits.

## Results

### Stability of the DraD invasin fimbrial subunit

#### DraD-sc does not possess a DraE-sc-like kinetic stability

To verify the kinetic stability of the DraD-sc protein we used a method that measures the resistance of a native protein structure to the unfolding caused by SDS, under condition of the SDS-PAGE electrophoresis [28]. Kinetically stable proteins, resistant to the SDS denaturation, incubated at moderate temperatures in the Laemmli buffer, demonstrate a gel retardation in comparison to an overheated protein sample. The analyzed DraD-sc protein samples were incubated at the temperature gradient from 25° to 100°C in the Laemmli buffer for 10 minutes and separated electrophoretically. In all analyzed samples the DraD-sc protein migrated at the same level of a molecular weight ca. 16 kDa. No band retardation was observed in samples incubated at lower temperatures (data not shown). The identically constructed recombinant DraE-sc protein tested in the same experiment showed the retardation observed up to 70°C [13]. The previously described DraE-sc-ΔSS protein (double Cys to Ala mutant) totally lost resistance to the SDS denaturation [13]. This experiment clearly shows that the DraD-sc protein which contains the disulfide bond connecting the B and F strands, does not possess high kinetic stability, characteristic for the DraE-sc protein. This experiment additionally supports thesis that the presence of the disulfide bond connecting the A and B strands may serve as an unique strategy for the high kinetic stability generation of the Ig-like fold which appeared exclusively in many fimbrial subunits of the chaperone/usher type adhesive structures [13].

#### DraD-sc possesses low thermodynamic stability

**Figure 2:**
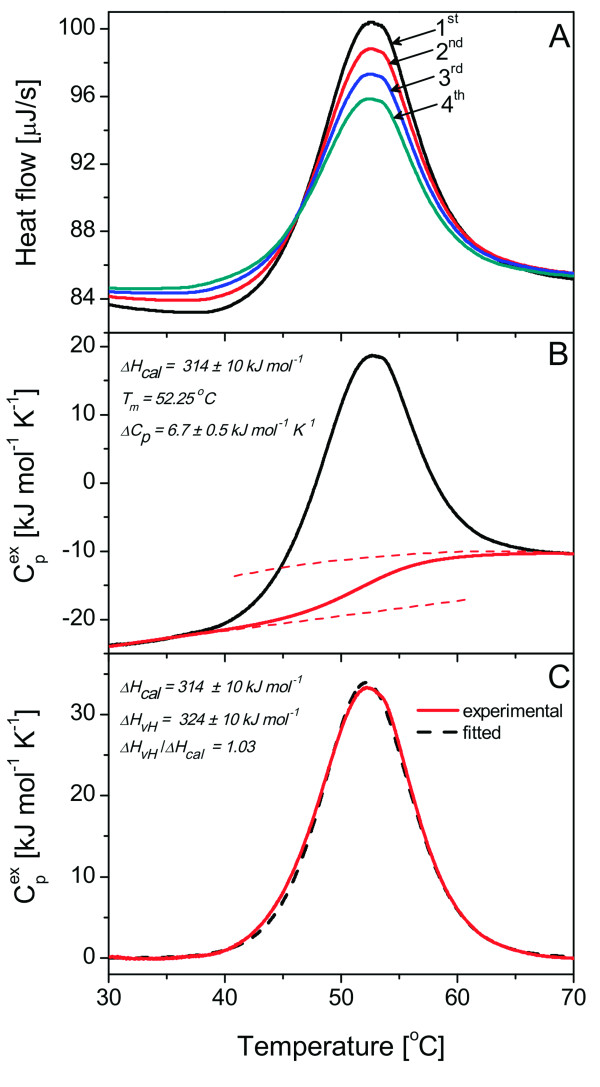
**Results of the DSC analysis of DraD-sc denaturation process**. **(A) **The raw calorimetric data representing the reversibility of the protein denaturation. Numbers denote the heating scans order. **(B) **The dependence of the excess heat capacity (C_p_^ex^) on temperature at scanning rate 1 K min^-1 ^before the baseline subtraction. **(C) **Experimental (red straight) and fitted (black dashed) DSC curves exhibiting high similarity.

To determine thermodynamic parameters and the mechanism of the DraD-sc protein thermal unfolding we used the most direct technique - differential scanning calorimetry (DSC). These experiments were performed using the same equipment and buffer conditions as in the case of the described DraE-sc and DraE-sc-ΔSS proteins [13,15]. This permitted us to conduct a reliable comparison of thermodynamic parameters characterizing these proteins. The DraD-sc protein unfolded reversibly with the temperature of transition T_m _of 52.25°C and the enthalpy of transition ΔH_cal _of 314 ± 10 kJ mol^-1 ^(Figure 2B). Even if the sample was overheated to 75°C the reversibility in the reheating scan was 91%. The posttransitional baseline was stable and was not affected by any detectable thermal effects of the aggregation phenomena (Figure 2A). Also, the calculated cooperativity ratio ΔH_vH_/ΔH_cal _equaled to 1.03 confirmed a high transition reversibility and allowed us to use the equilibrium two-state model (Figure 2C). The curvature of the transition and the baseline stability permitted also for a direct determination of the molar heat capacity change of the DraD-sc unfolding (ΔC_p _= 6.7 ± 0.5 kJ mol^-1 ^K^-1^), which corresponded to a ΔC_p _per mole of residues of 44.7 J mol^-1 ^K^-1^. Using all experimentally determined values of T_m_, ΔC_p _and ΔH_cal _we calculated the unfolding Gibbs free energy ΔG_T _and the unfolding enthalpy ΔH_T _at 25° and 37°C according to the following equations:(1)(2)

Where ΔH_Tm _is the reference enthalpy of transition, corresponding to the T_m_, the reference temperature, ΔC_pTm _is the difference between heat capacities of denatured and native state of protein at the reference temperature. The obtained calorimetric ΔG_25(37)°C _of 18.4 (12.3) ± 1.4 kJ mol^-1 ^correlates well with a published value of 17.9 ± 6.8 kJ mol^-1 ^at 25°C determined for the AfaD-sc protein using guanidinium chloride equilibrium denaturation experiments [14]. The presented standard unfolding free energy means that the stability of the DraD-sc invasin is rather low and it is close to the lower stability limit of 20 kJ mol^-1 ^typically observed for proteins collected in the ProTherm thermodynamic database [29]. The DraD-sc unfolding is also denoted by rather low enthalpy change ΔH_25(37)°C _of 131 (214) ± 25 kJ mol^-1 ^which is a consequence of structural distortion mainly in the region of the acceptor cleft. The presented data clearly showed that the low stability of the DraD-sc invasin contrasted with the data published so far concerning the stability of adhesive organelles of the chaperone/usher type and protein subunits.

### Formation of disulfide bond is necessary for DraD-sc folding

**Figure 3:**
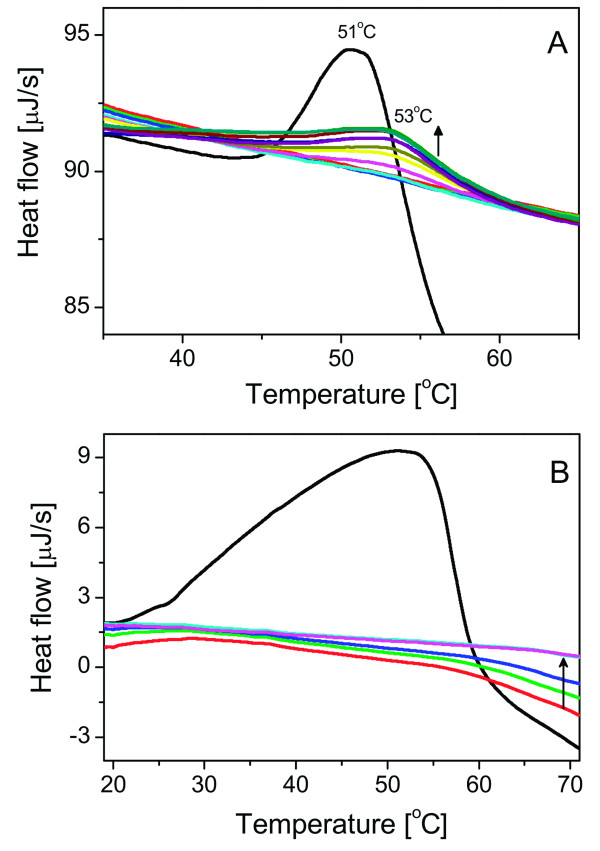
**Results of DSC experiments concerning the DraD-sc denaturation in reducing conditions**. **(A) **The denaturation in presence of 1 mM DTT in the protein sample. The first scan (black) is denoted with melting temperature 51°C. Next scans are denoted with melting temperature 53°C and increase in their intensity (marked with an arrow pointing up). **(B) **The denaturation in presence of 1 mM TCEP-HCl in the protein sample. The first scan is marked with black line, the rest is shown in color. First three re-heating scans exhibit different level and stabilize after the third re-scan (dark blue).

The DraD-sc protein possesses a disulfide bond connecting B and F strands that is uniquely localized compared to the other fimbrial subunits [13]. To examine the role of a disulfide bond in the DraD-sc structure we constructed an *E. coli *BL21(DE3)/pET30b(+)/DraD-sc-ΔSS strain encoding a mutant DraD-sc protein in which two cysteine residues were exchanged to alanine. This construct is identical to the previously described DraE-sc-ΔSS protein that was used to explore the influence of disulfide bond on adhesin subunit stability [13]. The DraD-sc-ΔSS invasin was not detected in the culture samples with monoclonal anti-His and polyclonal anti-DraD antibodies during the Western-blotting analysis. To reduce a potential proteolysis of the mutant invasin the bacterial cultures were cultivated at 30°C with IPTG as an expression inductor with a concentration of 0.25 mM. As a positive control of expression the *E. coli *BL21(DE3)/pET30b(+)/DraD-sc strain was used, which produced the disulfide containing DraD-sc. To examine the influence of the disulfide bond on the DraD-sc protein stability we planned a calorimetric experiment in which we used the DraD-sc sample with reduced disulfide bond as an effect of protein incubation in a buffer containing dithiothreitol (DTT). The efficiency of DraD-sc reduction was confirmed with Ellman's reaction that detects specifically free sulfhydryl groups. All final dialyzed calorimetric samples of the reduced DraD-sc contained 0.5 or 1 mM DTT to inhibit potential oxidation reactions. In the first heating DSC scan we observed a single endothermic peak with transition temperature of 51.8°C corresponding to the unfolding of reduced DraD-sc (experiment with 1 mM DTT in a buffer) (Figure 3A). After this transition there was a large visible exothermic effect connected with the protein aggregation phenomena. In the next four reheating scans the observed thermograms were rather flat without any endothermic peaks that might suggested that the DraD-sc refolded to some degree during cooling steps. In next five reheating scans (from 6 to 10) we observed an endothermic peak exhibiting a maximum at ca. 53°C (Figure 3A). The area of this peak increased in the following cooling/heating steps approaching in the fourth scan ca. 35% of the initial denaturation peak. At the same time, the heat capacity value of the sample decreased in the pretransition region of these scans that correspond to native protein. This effect could be connected with a slow regaining of the native structure in the next few steps.

We also examined the influence of the DTT at concentration of 0.5 mM on the DraD-sc calorimetric denaturation (data not shown). In this experiment the observed endothermic peak in the first heating scan was identical to the previously described. In the next scans peaks increasing in area with transition temperature of 53°C were observed at once, there was no thermograms without endothermic peak characteristic for experiments performed in buffer with 1 mM DTT. The peak with transition temperature of 53°C corresponded to the DraD-sc protein with a disulfide bond. This was confirmed with Ellman's test that detects free sulfhydryl groups by analysis of the soluble protein samples recovered from calorimeter cells after the end of experiment. The observed phenomena of the oxidized invasin appearance was connected with a well known thermal oxidative instability of DTT during calorimetric experiments [30]. DTT in heating scans was heated up to 75°C with scanning rate 1°C min^-1^, and after finishing each heating cells of calorimeter were equilibrated for 10 minutes at 75°C before cooling. The platinum cells of calorimeter may work as catalyst of the DTT oxidation. According to these data the re-oxidation of the DraD-sc began in calorimetric cell when the redox potential of a buffer dropped to a level sufficient for a disulfide bond formation. This explained observed differences in denaturation experiments performed in buffers with 0.5 and 1 mM of DTT. Additionally we performed analogical calorimetric denaturation experiments of DraD-sc invasin in buffers that contained TCEP-HCl instead of DTT. This disulfide reducing compound is resistant to oxygen oxidation, hence its concentration and reduction potential in the following reheating scans is stable [31]. In the first heating scan of the DraD-sc in a buffer containing 1 mM TCEP a huge endothermic peak was observed (Figure 3B). This was an effect of a few processes occurring during analysis. TCEP-HCl is a highly hydrophilic compound containing three carboxylic groups and, in contrast to DTT, is not able to reduce disulfide bonds that are buried in a hydrophobic protein core. So, the observed distortion of the DSC peak was an effect of an interaction of TCEP with the DraD-sc accompanied by a protein unfolding and a subsequent reduction of the disulfide bond. Next scans did not possess any endothermic peaks corresponding to the DraD-sc refolding (Figure 3B). The samples after experiments contained a fully reduced and aggregated DraD-sc protein.

The presented data clearly showed that the reduced DraD-sc was stable and unfolded at temperature only 1.5°C lower than the oxidized DraD-sc. Although only the melting temperature could be determined, and none of the thermodynamic parameters could not, it was that a disulfide bond only slightly stabilized the DraD-sc in its final structure. The observation that the refolding of the reduced DraD-sc during calorimetric experiments is strictly dependent on its re-oxidadion suggested that the formation of disulfide bond is crucial at some folding stages. This observation might explain the lack of the DraD-sc-ΔSS expression in which two cysteine residues were exchanged to alanine. In contrast to the invasin subunit, the mutation of cysteine residues to alanine in DraE-sc protein resulted in a higher level of expression than in the case of the protein containing a disulfide bond. The thermal denaturation of tha DraE-sc-ΔSS was almost totally reversible process in contrast to the reduced DraD-sc protein. The stabilizing effect of the disulfide bond on a final structure of the DraE-sc was significant and was probably responsible for observed high kinetic stability of that adhesin [13].

### FT-IR analysis of the DraD-sc thermal unfolding

To correlate determined calorimetrically stability parameters and mechanism of DraD-sc thermal unfolding with corresponding structural changes we analyzed this transition using the FT-IR spectroscopy. This technique permits to observe changes in protein secondary structure through monitoring C = O stretching vibration of the polypeptide backbone. The second derivative of a native secondary structure of the DraD-sc exhibits shape characteristic to Ig-like fimbrial proteins, similar to the previously observed [13,15]. A strong minimum at 1637 cm^-1 ^indicates that β-sheets are the main component of the folded structure. A small minimum at ca. 1660 cm^-1 ^suggests that some small turns are present too. The absence of other minima characteristic to other well known secondary structures allows us to conclude that the DraD-sc construct folds properly and its structure is consistent with a known crystallographic structures of the Dr fimbriae structural proteins.

**Figure 4:**
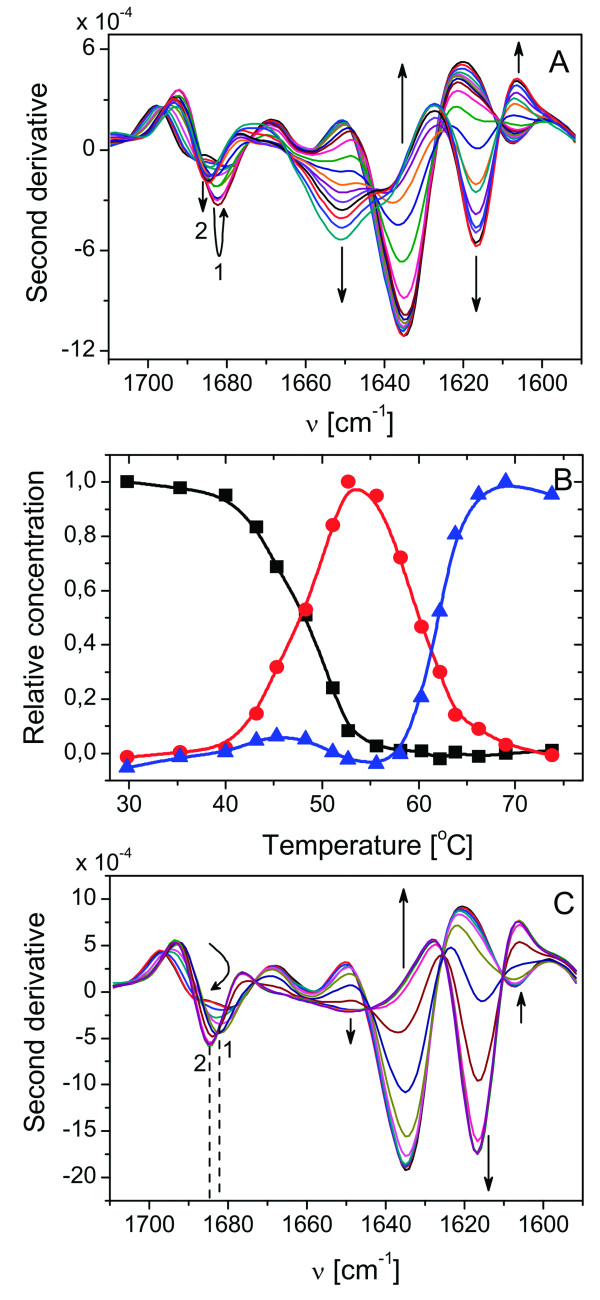
**The FT-IR results of the DraD-sc denaturiation experiments**. **(A) **Second derivatives of the DraD-sc at temperature range of 29.8 - 73.8°C. Numbers 1 and 2 denote the first and the second feature of two distinguishable transitions. Arrows indicate changes in minima intensities with an increase of temperature. **(B) **The result of chemometric analysis of denaturation data from the figure A. Three individuals are present (indicated with different colors and symbols), and two transitions are visible near 50°C and 60°C. **(C) **Second derivatives of the DraD-sc in the presence of 10 mM DTT at temperature range of 29.1 - 70.1°C. Description is the same as in figure A.

Although the structure is quite similar to the structure of previously characterized DraE-sc, the denaturation mechanism seems to be different [15]. The behavior of secondary structures observed in the infrared is not so straightforward, as in the case of the DraE-sc. There are no isosbestic points visible in the Figure 4A indicating that the denaturation mechanism cannot be simply described as two-state in conditions of the FT-IR experiment. Especially a region above 1680 cm^-1 ^suggests that at least two phenomena can be responsible for observed changes. To check this hypothesis, we employed the Factor Analysis [32]. The Principal factor analysis algorithm performed on the series concludes that three species are present during the presented temperature range (Figure 4B). Using the automated window factor analysis algorithm we can conclude that the first process occurs at a temperature ca. 50°C. This transition may be connected with the proper denaturation of the DraD-sc and may correspond to the transition denaturation peak visible in the DSC experiment (Figure 2). However, the second transition (above 60°C) is marked with strong changes in the secondary structure, mainly an aggregation characterized by a relatively strong minimum at 1618 cm^-1 ^and a small one at about 1684 cm^-1^. In the case of this protein, the process of denaturation is clearly separated from the aggregation process.

A series of second derivative spectra of the DraD-sc in the presence of DTT is very similar to the series of the DraD-sc itself (Figure 4C). The DTT does not affect the secondary structure of DraD-sc at low temperatures and it looks like it does not affect the first process attributed to the denaturation of the DraD-sc. Still two processes are distinguishable on the basis of the high wavenumbers region, however, they are almost simultaneous and it is difficult to say what is the exact temperature of the first process. The first process is accompanied by a second process of a massive aggregation, marked with a very strong minimum at 1618 cm^-1^. The denaturation temperature is not affected noticeably by the DTT presence, and the occurrence of isosbestic points, which suggest that only one resultant transition takes place, may indicate that it is similar as the previously described (i.e. ca. 50°C).

In contrast to other proteins we work on in our laboratory the process of DraD-sc denaturation (without DTT) is in some extent reversible in conditions of the FT-IR experiment. This may be stated on the basis of the location of the amide I' maximum (data not shown), which returns to the initial position of 1637 cm^-1^. However, the protein recovery is not complete, probably due to high concentration of the protein used in this type of experiment (ca. 20 mg ml^-1^), and characteristic aggregation bands are still visible in the spectrum of the DraD-sc. In the case of experiments concerning the usage of DTT there is no proof of any renaturation in the cooling step right after the heating.

The results of the analysis of FT-IR denaturation series spectra are quite consistent with the DSC results. The reversibility of denaturation and the significant separation of denaturation and aggregation steps are common features of the DraD-sc denaturation in conditions of both these experiments. Presented results of FT-IR experiments suggest also that the DTT does not affect the denaturation step of the DraD-sc. The presence of DTT plays a crucial role at the aggregation step, lowering its temperature from ca. 60°C to ca. 50°C. These two processes, namely denaturation and aggregation of protein in the presence of DTT, are hardly possible to be separated both in FT-IR and DSC experiments. Aggregation-characteristic features of DSC thermogram and FT-IR spectrum appear immediately after denaturation step (Figures 3A and 4C), and in both cases prevent from the direct analysis of the step. Although conditions of these experiments were different, they shed some light on the mechanism of the DraD-sc denaturation.

### Differences between DraD/AfaD and DraE/AfaE proteins core packing and donor strands adjustment

To describe quantitatively potential structural marks of the experimentally determined stability of the DraD-sc protein we calculated the shape correlation statistic (S_c_) [33] and the protein density parameter (PD) [34]. These two indexes describe the geometry of strands adjustment and the quality of the protein core packing, respectively. To better emphasize specific properties of the DraD-sc structure we compared it with that of the DraE/AfaE-sc. The most apparent difference between DraD-sc and DraE-sc proteins (and fimbrial subunits generally) visible in the structures can be noticed in the area of the acceptor cleft formed between the A and F strands [14,25]. Since these self-complemented structures are distorted in the area of the linker, the following shape correlation statistics and hydrogen bonds analysis are performed only for the last ten residues of the donor strands. In the AfaE-sc protein the donor G_d _strand is locked in the cleft by the total number of 10 backbone hydrogen bonds to both strands forming the complete seven stranded β-barrel structure (Figure 1B). The geometrical fit between the complementing donor strand and the acceptor cleft in the AfaE-sc is demonstrated by the calculated S_c _parameter of value 0.768. For the DraD protein the acceptor groove is distorted in such a way that the donor strand is able to form only 4 hydrogen bonds with the F strand only (Figure 1B). This correlates with the value of S_c _parameter for the acceptor cleft/donor strand interface of only 0.666. This comparison clearly showed that the adjustment of G_d _donor strand in the DraD-sc structure is very poor.

The difference in the protein density (PD) index observed in DraD-sc and AfaE/DraE-sc proteins, gives also some interesting information. Although the DraD-sc β-barrel structure is defective, in effect of imperfect interaction of G_d _donor strand with the acceptor cleft, the whole protein is characterized by a better core packing than in the AfaE-sc, the PD value of 0.741 and 0.709, respectively. The difference in protein packing between the DraD-sc and AfaE/DraE-sc is well visible in the region of so called tyrosine corner identified in both proteins at the beginning of the F strand with the tyrosines 108 in DraD and 112 in the DraE occupying equivalent positions (Figure 1C). The tyrosine corner is a common feature usually stabilizing the Greek key β-barrel structures which can be found [35,36]. In this motif the tyrosine side chain OH group makes a hydrogen bond to the backbone NH or CO moieties of residues Y-3, Y-4 or Y-5 with the middle case being the most common. Amino acids preceding the Tyr108 group in the DraD (the corresponding sequence is IAPGEY) match the Tyr corner consensus sequence which is LxPGxY. In the DraD subunit the tyrosine side chain's hydroxyl is positioned in the proximity of Ala104 CO group forming the most "classic" Δ4 tyrosine corner. The side chain of the Ile103 is buried in the strictly hydrophobic protein interior participating in forming of the tight hydrophobic core of the protein (Figure 1C). As for the DraE protein the Tyr112 and preceding residues (the corresponding sequence is TPPGNY) can be identified as the tyrosine corner motif. Interestingly, although the conformation of the Tyr112 side chain is very similar to that of Tyr108 in DraD, its OH group does not create any hydrogen bonds neither to the backbone CO nor NH groups of the preceding residues. That is the result of a slightly different conformation of this part of the backbone including the partially exposed Thr107 resembling the Ile103 in DraD and worse packing of the neighboring residues accommodating completely buried Arg16 residue (Figure 1C). Usually burial of a polar residue has destabilizing effect on the protein structure unless the buried residue maintains favorable interactions in the protein interior. The guanidinium moiety of Arg16 residue can form cation-pi interactions with Tyr112 phenyl ring, hydrogen bonds with backbone carbonyl of Lys14, side chain carbonyl of Asn106, and with backbone carbonyl of Leu140. The later residue belongs to the donor strand thus its interaction with Arg16 additionally anchors the G strand in the acceptor groove. On the other hand, when the donor strand is missing, Arg16 residue lacks two hydrogen bonds and thus its burial has additional destabilizing effect on the DraE subunit structure.

## Discussion

Fimbrial subunits form conserved protein family with Ig-like topology characterized by a missing of the seventh G strand which generates an opened hydrophobic groove in the structure. In the functional adhesin organelle the acceptor cleft is completed by the specific N-terminal donor strand G_d _of the neighboring subunit. Complementation of the groove by the donor strand results in formation of a very stable β-barrel with an optimized hydrogen bond network between strands and a well packed protein core. This general picture of fimbrial subunits contrasts with properties of the DraD/AfaD invasin subunit. The G_d _strand of DraD-sc is hydrogen bonded only with the F strand of the groove that results in the 'unzipped' β-barrel structure. In the region of the acceptor cleft there are six main-chain donors and acceptors of hydrogen bonds that do not participate in hydrogen bonds formation (Figure 1B). The worse fitting of the DraD-sc donor strand inside the cleft in relation to AfaE-sc protein is indicated by the S_c _value of 0.666 and 0.768, respectively. The published S_c _value of 0.78 for packing of specific G_d _donor strand in acceptor cleft of the Caf1 subunit of *Y. pestis *correlates well with that of the AfaE-sc. The S_c _parameter calculated for the interface between the Caf1 acceptor cleft and the G1 donor strand of the chaperone Caf1M is slightly lower and values of 0.76 [4]. This comparison emphasized the level of DraD structure distortion in the region of acceptor cleft. The structural destabilization of the DraD-sc protein is reflected in the standard Gibbs free energy of unfolding of only 18.4 ± 1.4 kJ mol^-1 ^that strongly contrasts with values obtained for the other self-complemented protein subunits of adhesive organelles: Caf1-sc 70-80 kJ mol^-1 ^(at 37°C) [5], type 1 pili subunits 50-80 kJ mol^-1 ^(at 25°C) [12] and AfaE-sc 45 kJ mol^-1 ^(at 25°C) [14]. This value is also lower than the standard ΔG of unfolding of defective structurally self-complemented subunits: DraE-sc-ΔSS (DraE without disulfide bond) 30 ± 5.0 kJ mol^-1 ^[13], DS_G_-FimH_P _(subunit with the parallel G_d _donor strand orientation) 26.5 ± 2.0 kJ mol^-1 ^and FimH_P_-DS_C _(subunit with the antiparallel G1 donor strand of the chaperone) 26.8 ± 2.0 kJ mol^-1 ^[12]. It is worth to note that native subunits missing the seventh β-strand are capable to fold but are marginally stable (ΔG_25°C _of unfolding of 8-10 kJ mol^-1^) [5,12,37]. The lower stability of the DraD-sc invasin is quite obvious when one compares its transition temperature T_m _with transition temperatures of DraE-sc and Caf1 subunits. The DraD-sc invasin denatures at 52.25°C, that is ca. 35°C lower then in the case of DraE-sc and Caf1, and 10°C lower then in the case of the DraE-sc-ΔSS [13,15]. The DraD-sc unfolding is also denoted by rather low enthalpy change ΔH_25(37)°C _of 131 (214) ± 25 kJ mol^-1^. This value is significantly lower than the one reported for the Caf1'' subunit, i.e. ΔH_37°C _395 kJ mol^-1^, which is close to the maximum observed for a globular proteins [5]. A direct comparison of the standard unfolding enthalpy of the DraD-sc to that of the DraE-sc is impossible because of irreversibility of calorimetric transition of the latter and its strictly kinetic character. However, the DraE-sc-ΔSS mutant, which is undistinguishable from DraE-sc on the basis of FT-IR spectra unfolds with the enthalpy ΔH_37°C _of 333 ± 20 kJ mol^-1^. In the case of the Caf1'' the observed melting enthalpy is a demonstration of almost perfectly optimized interactions that stabilize this protein. The low melting enthalpy of the DraD-sc is to a large extent a consequence of a structure distortion caused by a disorder of the hydrophobic core and the hydrogen bond network in a region of acceptor groove of the β-barrel (Figure 1B).

The another special feature of the DraD invasin is a localization of the disulfide bond that connects B and F strands of opposite sheets forming the β-barrel. This contrasts with a general position of the disulfide bridge in fimbrial subunits of the chaperone/usher system that joins neighboring A and B strands in the same sheet (Figure 1A) [13]. The presented data show that the disulfide bond is necessary at the stage of the DraD-sc folding as suggests the impossibility of obtaining an expression of the double Cys to Ala DraD-sc protein mutant. This disulfide dependent folding thesis is supported by calorimetric unfolding experiments of the reduced DraD-sc protein in a buffer containing reducing compound DTT and by FT-IR observation of the protein unfolding in the presence of this reducing agent.

The depicted unique structural features of the DraD-sc Ig-like fold and presented data on its thermal denaturation mechanism and stability properties determined by the DSC calorimetry and FT-IR spectroscopy differ with the properties of the DraE adhesin and generally fimbrial subunits of chaperone-usher type. The individuality of DraD/AfaD-like proteins is also reflected in its phylogenetic relations to the other fimbrial subunits. These invasins belong to a family of subunits assembling adhesive structures of γ3 clade [38]. This clade encompasses fimbrial structures encoded by the gene clusters of the FGL chaperone subfamily. However, the invasins form the individual subfamily within γ3 subunits clade characterized by a specific conserved domain PF05775 (Pfam, protein families database).

## Conclusions

The biogenesis of the chaperone-usher type adhesive structures is conserved process dependent on a common properties of fimbrial proteins: the chaperone dependent folding and the usher dependent surface cell localization in the form of a polymeric structure. This general view highly contrast with properties of DraD/AfaD invasin. 1) The chaperone independent folding - the DraD production is stable and fold properly in the periplasm of bacterial strains that do not co-express the *draB *chaperone gene [39]. 2) The usher independent surface localization - the DraD is transported via the type II secretor [40]. 3) The DraD may occur at the cell surface as an unbound subunit with the open acceptor cleft or loosely attached at the tip of Dr fimbriae [22,27]. 4) Although it is a potential tip subunit it is not required as an initial subunit in the biogenesis of Dr fimbriae [39]. Despite available structural data the molecular sources of these unique properties are poorly determined. Presented in this paper data permit to precise two speculative thesis. 1) The protein expression, DSC microcalorimetry and FT-IR spectroscopy *in vitro *experiments showed that the disulfide bond unique to the DraD is crucial to its proper folding. This render a question: Is the formation of the specific to the DraD/AfaD disulfide bond crucial to the observed chaperone independent invasin folding? 2) The structural comparison of the DraD/AfaD invasin with the DraE and other fimbrial subunits emphasize its structural optimization outside the region of the acceptor cleft. This is denoted by the protein density parameter and some minor structural elements such as tyrosine corner. This also render a question: Are the disulfide bond and described minor structural elements responsible for the observed stability of the DraD in the form with the open acceptor cleft? Interestingly, the DraD-like SafD and SefD invasins that are initiator subunits of the *S. typhi *and *S. enteritidis *pili biogenesis, respectively are transported to the cell surface via the classical chaperone/usher pathway do not possess any disulfide bond in their structures. This additionally confirms the thesis that the unique disulfide bond of the DraD enables its folding and the usher independent transport to the cell surface.

The presented in this work calorimetric and FT-IR spectroscopy data described the stability and mechanism of thermal denaturation of the self-complemented form of DraD - the minimal model of invasin protein in the fimbrial tip complex. This protein construction strategy was widely used in the studies of fimbrial subunits of the chaperone-usher type. This permitted us to perform a straight comparison between DraD-sc properties and that of other subunits and enabled to conclude that properties of DraD are unique in the context of its belonging to the family of fimbrial subunits. Although the above presented thesis concerning potential structural sources of unique biological properties of the native (with open acceptor cleft) DraD should be verified in future experiments based on the native DraD protein.

## Methods

### Protein expression and purification

DraD-sc (self-complemented) is a recombinant fusion protein composed of the following segments in N to C direction: the N-terminal signal peptide, the His_6_-Tag peptide AELHHHHHH connected with DraD β-sandwich complemented at C-terminus by the specific N-terminal donor strand GFTPSGTTGTTKLTVT of the DraE protein inserted using linker peptide DNKQ. The native *draD *gene possesses the unique SacI site located immediately after sequence encoding signal peptide, similarly as in the case of the *draE *gene. This permits to use in construction of expression plasmid pET30-DraD-sc encoding DraD-sc an identical cloning strategy as in the case of the pET30-DraE-sc plasmid [15]: forward primer 5'-tatgagctccaccaccaccaccaccacGCTGAACTGCACCTGGAGAGCCGGGGAGGTTC-3' (SacI site is underlined, the sequence coding His-Tag is lower case, the complementary sequence is upper case) and reverse primer 5'-tataagctttcaggtaacggtcagtttggtggtaccggtggtgccagacggggtgaaaccctgtttgttgtcTTCCTGTGGCACCACACAGGCTCCGCCAACCG-3' (the HindIII restriction site and stop codon are underlined, the sequence encoding linker peptide and donor strand is lower case, the complementary sequence is upper case). Expression plasmid pET30-DraD-sc-ΔSS encoding DraD-sc, the disulfide bond lacking mutant, was created on the basis of pET30-DraD-sc plasmid using PCR borne Site Directed - Mutagenesis Kit (Stratagene, CA, USA) to change codons encoding Cys to Ala. The DraD-sc protein was produced in *E. coli *BL21(DE3)/pET30-DraD-sc strain using the same procedure as in case of the DraE-sc protein [15]. The *E. coli *BL21(DE3)/pET30-DraD-sc-ΔSS culture was grown with an agitation at 30°C to OD_600 _= 0.3, induced by an adding IPTG to a final concentration of 0.25 mM and grown for an additional 2 h. The DraD-sc invasin was purified from isolated periplasmic fractions by Ni^2+ ^- affinity chromatography on IMAC Sepharose 6 Fast Flow resin (GE Healthcare Bio-Sciences AB, Uppsala, Sweden) and exclusion chromatography on a Superdex 75 10/300 GL column (Amersham Biosciences, Uppsala, Sweden) using procedure as in case of the DraE-sc [15].

### Microcalorimetry and other techniques

DSC experiments were performed on the CSC 6300 Nano-DSC III differential scanning microcalorimeter (Calorimetry Sciences Corporation, Lindon, UT, USA) with the capillary cell volume of 0.299 ml at the temperature range from 10 to 75°C. The experimental data were recorded using DSCRun software (Calorimetry Sciences Corporation, Lindon, UT, USA). The concentration of DraD-sc (molecular mass 16.34 kDa) were ca. 1.75 mg ml^-1 ^in each experiment. The analysis was performed with the scanning rate of 1°C min^-1^. Samples preparation, measurements and data analyses were performed identical as described previously for DraE-sc and DraE-sc-ΔSS proteins [13,15]. The DraD-sc protein (disulfide containing) was analyzed calorimetrically in a buffer: 20 mM sodium phosphate, pH 7.5, 100 mM NaCl. To prepare DraD-sc protein samples with the disulfide bond chemically reduced by DTT (dithiothreitol) the following procedure was used. The DraD-sc protein was dialyzed to a buffer: 20 mM Tris-HCl, pH 8.0 and 100 mM NaCl. To the dialyzed sample DTT was added to the final concentration of 30 mM. Reduction of disulfide bonds was conducted 4 h at the room temperature with gently agitation and than continued overnight at the temperature of 4°C. After reduction reaction the protein sample was dialyzed to a buffer: 20 mM sodium phosphate, pH 7.5, 100 mM NaCl and 0.5 or 1 mM DTT as an antioxidant. The reduction of DraD-sc protein with TCEP-HCl (Tris(2-carboxyethyl)phosphine HCl)) was performed as follows. The DraD-sc protein was dialyzed to buffer: 50 mM sodium citrate, pH 5.0, 80 mM NaCl after this the TCEP-HCl was added to the final concentration of 30 mM. The reduction process was performed identically as in case of the DTT. After reduction stage the sample was dialyzed to a buffer: 50 mM sodium citrate, pH 5.0, 80 mM NaCl and 1 mM TCEP-HCl. The detection of sulfhydryl groups in the DraD-sc protein samples were performed with Ellman's reagent (DTNB, 5,5'-Dithio-*bis*-(2-nitrobenzoic acid)). Before analysis the protein samples were rapidly dialyzed to a detection buffer: 100 mM sodium phosphate, pH 8.0, 1 mM EDTA, using Amicon Ultra concentrator (Millipore, Bedford, MA, USA). To the 250 μl of protein samples the 50 μl of DTNB (4 mg/ml in the detection buffer) was added. After 15 minutes of incubation at the room temperature the sample absorbencies were measured at 412 nm. Calculations were performed using molar extinction coefficient of TNB of 14.150 M^-1^cm^-1^. The FT-IR spectroscopy and SDS-polyacrylamide gel electrophoresis assay were performed as described previously for DraE-sc and DraE-sc-ΔSS proteins [13,15]. The protein concentration in the FT-IR experiments was ca. 20 mg ml^-1^. The Factor Analysis was performed using the Matlab R2010a program (The MathWorks, Natick, MA, USA).

### Assessing the structural features

Packing density (PD) is a measure of the quality of protein core packing. It is calculated as a fraction of the protein's solvent excluded volume occupied by its Van der Waals volume. The value of this parameter varies between 0 and 1 and for proteins with perfect core packing PD value approaches the later value. Although the average packing of the protein interior depends on its size with smaller molecules being better packed on average, for molecules of similar sizes packing density is a good measure of their internal fit. For PD calculations we used procedure as described [34]. To check the geometrical fit between the complementing donor strand and the acceptor cleft shape correlation statistics (S_c_) were calculated [33]. This parameter indicates how well two surfaces fit each other. Its value varies between 0 and 1 with the former value indicating no geometrical fit and the later one indicating the perfect fit of the two surfaces. The number of hydrogen bonds was calculated for the energy minimized structures of both subunits with the GROMACS tool g_hbonds [41]. The calculations were based on the AfaD/DraD (pdb code: 2fvn) and the AfaE (1rxl) structures deposited in the Protein Data Bank.

## Abbreviations

C_p_^ex^: the excess heat capacity; DraD the invasin subunit encoded by a *dra *operon; DraD-sc: the self-complemented DraD subunit; DraD-sc-ΔSS: the disulfide bond lacking mutant of DraD-sc; DraE: the adhesive subunit of Dr fimbriae; DraE-sc: the self-complemented DraE subunit; DraE-sc-ΔSS: the disulfide bond lacking mutant of DraE-sc; DSC: the differential scanning calorimetry; ΔC_p_: the heat capacity change of protein unfolding; ΔG: the free energy change of protein unfolding; ΔH: the enthalpy change of protein unfolding; FT-IR: the Fourier transform infrared spectroscopy; T_m_: the protein melting temperature.

## Authors' contributions

RP carried out the DSC microcalorimetry, the SDS-PAGE kinetic stability experiments and drafted the manuscript. PB carried out the FT-IR spectroscopy experiments, participated in analyzing DSC data, drafted the manuscript. BZP constructed DNA plasmids and drafted the manuscript. MW carried out structural and computational analyses, drafted the manuscript. JN carried out protein purification and drafted the manuscript. JK drafted the manuscript. All authors read and approved the final manuscript.
